# Real-Time Safety Optimization of Connected Vehicle Trajectories Using Reinforcement Learning

**DOI:** 10.3390/s21113864

**Published:** 2021-06-03

**Authors:** Tarek Ghoul, Tarek Sayed

**Affiliations:** Department of Civil Engineering, University of British Columbia, 6250 Applied Science Lane, Vancouver, BC V6T 1Z4, Canada; tarek06@mail.ubc.ca

**Keywords:** trajectory optimization, dynamic speed advisory, real-time safety, connected vehicles, reinforcement learning

## Abstract

Speed advisories are used on highways to inform vehicles of upcoming changes in traffic conditions and apply a variable speed limit to reduce traffic conflicts and delays. This study applies a similar concept to intersections with respect to connected vehicles to provide dynamic speed advisories in real-time that guide vehicles towards an optimum speed. Real-time safety evaluation models for signalized intersections that depend on dynamic traffic parameters such as traffic volume and shock wave characteristics were used for this purpose. The proposed algorithm incorporates a rule-based approach alongside a Deep Deterministic Policy Gradient reinforcement learning technique (DDPG) to assign ideal speeds for connected vehicles at intersections and improve safety. The system was tested on two intersections using real-world data and yielded an average reduction in traffic conflicts ranging from 9% to 23%. Further analysis was performed to show that the algorithm yields tangible results even at lower market penetration rates (MPR). The algorithm was tested on the same intersection with different traffic volume conditions as well as on another intersection with different physical constraints and characteristics. The proposed algorithm provides a low-cost approach that is not computationally intensive and works towards optimizing for safety by reducing rear-end traffic conflicts.

## 1. Introduction

The market share of connected vehicles (CVs) has drastically increased in recent years, with multiple projections estimating that the majority of vehicles produced by 2045 will have CV capabilities [1]. CVs present an opportunity to optimize road networks for safety and mobility in real-time. By utilizing vehicle-to-vehicle (V2V), vehicle-to-infrastructure (V2I), and infrastructure-to-vehicle (I2V) communication systems, connected vehicles provide situational awareness to both the infrastructure that they utilize and to the vehicle users themselves.

The implementation of CV-related control technologies at intersections appears to be a promising field of research. Intersections, specifically during phase changes, represent a large proportion of vehicle collisions, with 45% of collisions in British Columbia occurring at intersections [2]. Rear-end collisions, in particular, make up a significant portion of these collisions and are to be examined in this paper. Collision risk factors and road user behavior at intersections have been extensively studied, with several conflict-based Safety Performance Functions (SPFs) and metrics being devised to predict and reduce traffic conflicts and collisions [3].

Various studies have attempted to apply data from connected vehicles to optimize safety and delay-related performance metrics using V2I data. Most notably, applications of V2I CV data include the use of Adaptive Traffic Signal Controllers (ATSCs), which seek to optimize traffic by modifying signal timing. These applications have been applied both to individual intersections [4,5,6,7] and entire roadway networks [8].

For safety applications and optimization, signal timing is easier to modify than the behavior of individual vehicles, especially vehicles that lack connectivity capabilities. As additional CVs are introduced to roadways, I2V methods of optimizing vehicular traffic by modifying driver behavior and vehicle trajectories have become more feasible. Examples of these techniques include eco-driving systems that modify trajectories through speed advisories such that vehicular emissions are minimized [9,10]. This framework may be expanded upon and combined with ATSC systems to develop Signal Vehicle Coupled Control systems (SVCCs), which modify both vehicle trajectories and signal timing to optimize for parameters such as delay and emissions [7,11].

Most trajectory optimization algorithms proposed in previous studies do not typically focus on traffic safety optimization in real time. Therefore, this paper seeks to develop a trajectory optimization algorithm for vehicles at intersections, using speed advisories, that fulfills the following criteria:

- The algorithm needs to be able to reduce traffic conflicts considerably in real-time;
- The algorithm should utilize readily available CV data that is straightforward to obtain;
- The algorithm should not be computationally intensive to ensure that the average roadside unit may be able to run the computations necessary for real-time analysis;
- The proposed system needs to consider conventional vehicles in the network as well as a mixed traffic environment;
- The proposed system must be resilient to sensor corruption and uncertainty;
- The system should be independent and transferable to existing and future CV-based ATSC systems.

## 2. Previous Work

### 2.1. Traffic Information Extraction

The accuracy of any model is usually dependent on both the technique used and the input parameters. Thus, accurate sensor technology is required to ensure that the proposed algorithm can collect data that reflects reality. Several studies have been performed regarding traffic information extraction using Vehicle to Infrastructure (V2I) and Vehicle to Vehicle (V2V) communication.

Dynamic traffic parameters such as shockwave characteristics are important metrics that may be used to develop traffic safety models. Numerous algorithms have been developed to identify these parameters. These algorithms include the estimation of queue length using two points through Bayesian Network modeling [12,13], the estimation of traffic parameters using GPS position data or mobile phone data [14,15] and estimating queue length using probe vehicle V2I communication systems [16,17]. While an abundance of techniques is available, according to a review by Guo et al. 2019, traffic information extraction via V2I and V2V can be categorized into three distinct categories; shockwave-based approaches, kinematic equation-based approaches, and stochastic learning approaches [6]. The literature suggests that by using any of these techniques, the presence of connected vehicles on the road and key traffic safety parameters can be obtained to a reasonably accurate degree through V2I and V2V capabilities in real-time data collection.

### 2.2. Real-Time Safety Evaluation

Real-time safety evaluation is necessary to develop any dynamic system or intervention to improve safety. Waiting for a statistically significant number of collisions to occur to identify safety issues and implement countermeasures is reactive and presents an ethical dilemma. The use of surrogate measures such as traffic conflicts can be used to evaluate safety in a more proactive approach. Traffic conflict measures such as Time to Collision (TTC), Post Encroachment Time (PET), and Deceleration Rate to Avoid Crash (DRAC), are commonly used as performance measures [18]. Modified versions of TTC and DRAC have also been devised to estimate collisions using different definitions of traffic conflicts [19,20].

Several studies have attempted to develop Safety Performance Functions (SPFs) in order to relate dynamic traffic parameters to conflicts and subsequently collisions at the cycle level. Data relating to shockwave parameters such as shockwave area and queue length have been used to evaluate safety at the cycle level, with the same measures being generalized to provide real-time safety measures [3].

### 2.3. Platoon Behavior

Platoons are defined as a group of vehicles moving along a specific approach, grouped based on several predefined parameters. Several techniques to designate vehicles as part of a platoon are available, such as reinforcement learning and dynamic programming-based approaches. Some studies classified vehicles as forming part of a platoon based on the headway of following and leading vehicles [21], while others utilized different thresholds, such as the number of vehicles within the group, density, or speed differentials, to establish similar states and group vehicles together as part of the same platoon [22,23,24].

Platooning behavior is used in multiple traffic signal optimization algorithms in order to improve safety and throughput [25]. This behavior could be applied to trajectory optimization by using similar techniques. Platoon formation or dissipation may influence the arrival pattern at a signal stop-line, which would have implications for signal optimization as platoons may be joined together or divided. By utilizing this idea and grouping vehicles together, ATSC systems may see further safety benefits [25] if used with the algorithm that this paper currently seeks to develop.

The presence of conventional vehicles presents a challenge for real-time safety optimization as not all vehicles can be expected to transmit data. Platooning behavior may allow for the use of pacer vehicles, which can be used to regulate the speed of conventional vehicles forming part of the platoon [26]. A study by Kreideh et al. 2018 noted that the use of autonomous vehicles in freeway environments with less than a 2.5% market penetration ratio (MPR) managed to greatly reduce the magnitude of stop and go waves, improving traffic stability [27].

### 2.4. Previous Vehicle Trajectory Optimization Algorithms

#### 2.4.1. Variable Speed Limits

This paper focuses primarily on the possibility of applying Variable Speed Limits (VSL) with short refresh rates to individual vehicles or groups of vehicles. VSLs have existed and been in operation since the 1960s [28]. The intent of VSLs is to assist in reducing the speed variance between upstream and downstream vehicles on highways, which typically results in instability and increased collision risk. By sending a signal to drivers, either through a roadside sign or directly to the vehicle via I2V communication, a specific span of roadway may change its speed limit based on the situation. This is known as homogenization, in which a critical speed is achieved that corresponds to the maximum flow, reducing variance and thus traffic conflicts [28]. Studies have been performed demonstrating the effectiveness of VSLs in specific situations such as in merging [29] and in reducing bottlenecks by modifying upstream speeds [30].

A major limitation with respect to VSLs and speed limits, in general, is the issue of compliance. This is examined in a case study in Prague that concluded that despite the imposition of a variable speed limit in a ring road, there was insufficient evidence to suggest that there was a significant difference in speed resulting from the imposition of VSLs. A larger variance in speed was observed due to compliant drivers moving onto the right lane and non-compliant drivers driving faster due to the lane change [31]. As a result, proper enforcement measures must be taken should VSLs be implemented. Additionally, it is also important to ensure that there is not a significant speed differential between the various lanes as the increased variance in speed would result in additional collisions. Furthermore, any devised algorithm must account for a significant proportion of non-compliant vehicles in the connected vehicle context, thus resulting in additional modeling being necessary. For the purposes of this study, a non-compliant vehicle may be treated as a conventional vehicle with respect to market penetration ratio modeling.

#### 2.4.2. Trajectory Optimization at Intersections

Several studies have considered the implementation of trajectory optimization techniques in intersections when used in conjunction with signal optimization. These studies typically apply to connected autonomous vehicles (CAVs) and operate by either supplementing the adaptive traffic signal control (ATSC) system to provide additional gains with regards to carbon emission and delay reduction [9,32] or work to establish a reservation system in which certain vehicles following a certain trajectory are provided with a window of time and space to clear the intersection [33,34].

The most commonly used trajectory optimization techniques with regards to CVs fall under the category of systems known as Green Light Optimal Speed Advisory (GLOSA) systems. Previous studies have developed GLOSA systems for both multiple-segment systems considering routing, as well as discrete signal segment localities. Multiple-segment systems according to the literature are more likely to provide improved performance provided that the traffic conditions are flexible enough to allow for a wide range of speeds over a larger distance [35]. Similar field studies relating to the length of roadway segments have shown that providing drivers with signal state data earlier yields improvements to reaction time and safety [36]. It is thus favorable to provide advisories as early as possible.

Studies involving the performance of GLOSA systems have shown promising results with benefits to fuel efficiency and delay reduction observed in high-density environments if applied appropriately [37,38]. GLOSA algorithms that were previously derived rely primarily on conditional logic that is either based on current vehicle position in designated stop and go zones, [37] or on trajectory prediction for discrete vehicles [38,39,40]. Certain more complex methods include the use of genetic algorithms fuzzy rule-based systems to reduce congestion over a longer span of roadway [41]. At the most basic level, GLOSA presents the concept of a time to intersection (TTI) and establishes a means by which a TTI threshold is calculated and a favorable slowdown speed is obtained.

The use of trajectory optimization with signal optimization is referred to as a Signal-Vehicle Coupled Control (SVCC). Studies have been performed on the topic but typically relate to delay or vehicular emissions [7,9,10,11] and understate the importance of the trajectory optimization process as it relates to safety. Furthermore, these studies rarely consider computational complexity and rely on techniques such as mixed-integer linear programming or dynamic programming approaches [7,42]. To the best of the author’s knowledge, this is the first study that seeks to optimize connected vehicle trajectories at intersections using speed advisories with respect to safety in real-time.

### 2.5. Reinforcement Learning Techniques

Reinforcement learning is a subset of machine learning that deals with a system or agent that interacts with a dynamic environment in order to optimize for a certain parameter or achieve a certain behavior. Unlike supervised learning, the agent in question learns iteratively through a Monte Carlo process in which a function, known as the reward function, is used to evaluate the effectiveness of previous actions and adjust the policy accordingly.

The optimization processes vary depending on the reinforcement learning algorithm used. The most basic of which is known as the Q-learning approach, which establishes a matrix that includes all possible states, i.e., conditions, as well as the outcome for every possible action given a current state. Simple Q-learning thus excels in simple situations where the state and action at any given time are discrete and few combinations of state-action pairs exist. Thus, by utilizing the Q-matrix, an objective function known as the Q-function can be optimized via Bellman’s Equation and the matrix may be filled. The process of filling the Q-matrix is known as exploration, in which the agent begins to learn the various environmental states and the outcome of various actions given a particular state. The selection of the optimum action given a current state is defined by the agent’s policy, which is defined by the Q-matrix and is refined over time. Q-learning, however, breaks down with continuous environments requiring the discretization of continuous data. It further breaks down when said continuous environments are large, complex, and feature a large number of different combinations resulting in a large combinatorics problem [43].

To remedy the issue with respect to the exponentially growing Q-matrix, alternative reinforcement learning techniques have been devised that may obtain the Q-value of any given state-action pair through an approximation of the Q-matrix rather than a lookup. The Deep-Q-Network technique (DQN) utilizes a neural network architecture to establish a model that estimates the Q-value of an action. The DQN agent uses a discrete state to output a discrete action or series of discrete actions. The issue with respect to DQN networks is that while it is possible to discretize a continuous observation state space with reasonable accuracy, it is not possible to output a continuous action. The action space of a DQN is defined by the number of output nodes, each corresponding with a single action. An action that involves three different variables would thus be mapped as a single node. Thus, every combination of the three action variables would have to have its own distinct action output node resulting in a large level of complexity, and thus decreasing convergence with every additional variable [44].

Alternative systems have been devised in order to remedy this issue, including the use of alternative agents such as Deep Deterministic Policy Gradients (DDPG) [45], Proximal Policy Optimization (PPO) [46], and Soft Actor-Critic networks (SAC) [47]. These algorithms make use of multiple agents rather than one agent and may also be used where the action space is continuous. This allows for multiple variables to be optimized without resulting in an exponential growth of output parameters and complexity. These agents differ from DQNs and simpler models in that they utilize networks involving an actor and a critic. The actor decides upon a given action and the critic estimates the reward for that action allowing for a more optimum policy to be determined.

## 3. Materials and Methods

This paper considers two signalized intersections with four approaches operating under normal conditions. The study intersections were created based on real intersections in Surrey, BC, and real-time video data were used to ensure that the model accurately represents reality. Utilizing key concepts from precedent work, a reinforcement learning approach was applied to the problem and simplified using dynamic programming. The algorithm used can be described as microscopic and uses basic kinematic relations in order to optimize for safety. Thus, a dynamic programming approach was used to identify situations that warrant the determination of an ideal speed. A reinforcement learning algorithm then computes and issues these ideal speeds using observation data from the environment to optimize a reward function that minimizes traffic conflicts.

### 3.1. Selecting a Function to Quantify Safety

**Figure 1:**
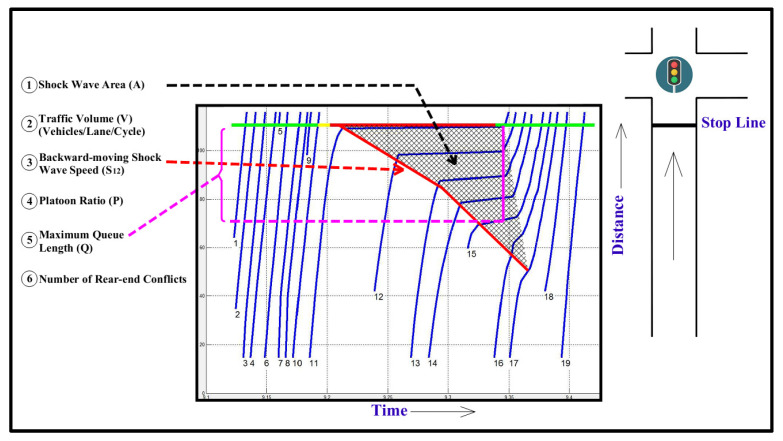
Shockwave parameters and traffic conflict models (Essa and Sayed 2018).

Essa et al. 2020 [48] developed several safety performance functions that can be used to quantify vehicular safety in short time intervals, which allows for traffic conflicts to be estimated at the cycle level in the ATSC context. The safety performance functions relate dynamic traffic parameters, such as shockwave characteristics and cycle parameters, to estimate traffic conflicts. The most significant parameter used was the shockwave area, which is strongly correlated with rear-end conflicts at intersections. The derived safety performance functions are as follows, with the input variables defined in Figure 1:

(1) $$E\left(Y\right)=V^{1.563}exp\left(-3.231\right)$$

(2) $$E\left(Y\right)=V^{0.706}exp\left(-1.797+0.501\;A\right)$$

(3) $$E\left(Y\right)=V^{0.65}exp\left(-2.046+0.0122\;Q\right)$$

(4) $$E\left(Y\right)=V^{1.637}exp\left(-3.316+0.05\;S_{12}\right)$$

(5) $$E\left(Y\right)=V^{1.571}exp\left(-1.768-1.266\;P\right)$$

(6) $$E\left(Y\right)=V^{1.239}exp\left(-1.624+0.294\;A-0.828\;P+0.119\;S_{12}\right)$$

Given that this paper does not seek to optimize signal timing, signal level parameters such as the platoon ratio may not be representative of the desired performance of the system. Ideal behavior would be characterized by vehicles slowing down to an ideal speed if they cannot clear an intersection or speeding up to clear an intersection, minimizing idle time at the intersection. Furthermore, for the purpose of real-time optimization, a measure that is easily obtained within seconds is preferable to one that requires the entire cycle to process or relies upon rolling averages. Thus, the most useful parameters used in this study are shockwave area and traffic volume, corresponding to the second formula derived in the previous study. The shockwave area can be obtained at any given point in time. Changes in shockwave area may be instantaneous, making it a useful measure for real-time safety evaluations. Similarly, traffic volume is among the simplest variables to obtain, and changes are easy to observe.

Therefore, the model in Equation (2) was selected as the primary performance measure. Since the intersection that will apply these measures includes multiple approaches, the total safety at the intersection must consider all approaches. Thus, traffic safety can be quantified using the sum of expected conflicts for a given lane and approach as defined by Equation (2). With an appropriate measure of safety clearly defined, this will be used to improve vehicle safety at the intersection.

It should be noted that the functions derived in the precedent studies only apply to rear-end conflicts. Given that these are the most common collisions at intersections, other conflict types, such as side swipes and left-turn conflicts, were not considered and may be examined in future research.

### 3.2. Platoon Recognition Algorithm

Given the large number of vehicles expected to be present in the system, organizing these vehicles into groups may be beneficial to simplify the problem. A review of the literature indicated that platoon identification is a contentious topic with complex methods used to identify clusters of vehicles in situations where more than one lane is present. In this paper, a simplified version of the platoon recognition algorithms found in the literature was used.

As observed in the literature, some techniques identify platoons based on the minimum number of following vehicles, while others utilize a critical headway parameter to designate vehicles forming part of a platoon [21]. After calibration and through trial and error, a critical minimum platoon size of three vehicles, a maximum critical time headway of 5 s, and a space headway of 35 m was selected. These variables were selected and calibrated through testing and validated by visual inspection of the results using simulation software. The methodology used was based on the key variable of critical time headway identified by Kamonthep et al. 2019 [21].

By utilizing this concept, platoons can be identified on single lanes. The framework works reasonably well when considering two separate adjacent lanes. During the calibration process, the observed likelihood of a cluster of vehicles on different lanes sharing the same longitudinal positions but being classified as two separate platoons was minimal. Nonetheless, it is necessary to account for this potential scenario should it arise. To modify this framework to adapt to multiple lanes, the first and last vehicles of the platoon on each lane are identified. Their positions are then transferred onto adjacent lanes, and the maximum of this range, provided that the headways do not exceed the critical values, was used to further classify the same platoon across multiple lanes. Thus, the position of the first and last vehicle of the first platoon in Lane A was compared to the position of the first and last vehicle of the first platoon in Lane B, as well as adjacent platoons to recalibrate and develop a system that may be used for two-lane approaches.

This approach was primarily used to ensure that the model remained simple and easy to implement in real-time. Noting the inherent limitations of using a fixed critical parameter assumed via calibration, the platoon recognition used for the purposes of this paper is simple and is not as accurate as other more complex methods. It would be possible to further refine this system by utilizing a supervised learning approach or by applying more complex pattern recognition algorithms. This system, however, remains accurate enough for the purposes of defining gaps between identified platoons of vehicles and allowing for a system to decide whether to close the gaps, create new gaps, and dynamically assign speeds to the various groups.

### 3.3. Visualizing the Ideal Individual Vehicle Behavior

It is important to identify the ideal vehicle behavior such that a solution that is grounded in theory can be found. In theory, the ideal discrete vehicle behavior would be defined based on the signal state, with the vehicle accelerating or decelerating depending on whether the vehicle can safely cross the intersection. Should the vehicle decelerate to a low speed, this may have negative effects on traffic flow and potentially create stop-and-go waves over time. Thus, in the event of a deceleration, there is a critical target speed that the algorithm should seek to obtain that would prevent additional shockwaves from being formed.

Furthermore, platooning behavior should be considered. Should a vehicle be asked to slow down, it would likely join with the preceding vehicles to form a platoon with the first leading vehicle regulating the speed of vehicles behind it. Vehicles asked to speed up may similarly join with vehicles ahead to increase the size of a given platoon. Individual vehicle behavior would compound into group behavior that would result in entire platoons forming, dissipating, splitting, or joining together.

Noting the integration between platoon group behavior and individual vehicle behavior, the ideal solution would be ultimately dependent on signal state and projected trajectory, allowing for a set of different solutions to be established and applied to entire platoons of vehicles.

#### 3.3.1. Scenario 1: Vehicle Arrives at Intersection during Green Phase

In the first scenario, a given vehicle may be expected to arrive and clear the intersection prior to the next signal phase, assuming that it maintains its current speed. In this scenario, the simple solution is to ensure that the vehicle accelerates to the maximum allowable speed in order to allow as many vehicles as possible to cross the intersection. Thus, this condition may be defined by the inequality:

$$t_{green\;remaining}>\frac{x_{vehicle}-x_{stopline}}{v_{vehicle}}$$

Should the inequality be false, then the vehicle is expected to arrive after the signal turns red. As such, assuming that the vehicle is unable to speed up to clear the intersection, it would be best for it to slow down in order to reduce the shockwave area. A signal may be sent to the connected vehicle in question, and it would be informed ahead of time that it cannot cross the intersection in time, allowing for more prudent driving behavior.

#### 3.3.2. Scenario 2: Vehicle Arrives at Intersection during Red Phase

In the second scenario, a vehicle may enter the sensor range during the red phase. If the vehicle is expected to arrive during the red phase, then it would be best for it to slow down ahead of time given the fact that the vehicle would have to wait at the intersection. This behavior would similarly reduce the shockwave area and result in a safer driving experience. The speed at which it slows down must be within a given threshold, as defined in the paper by Li et al. 2014 [7] as to not create additional shockwaves upstream. This scenario is thus given by the following inequality after which a “slow down speed” must be provided to the given vehicle:

$$t_{red\;remaining}>\frac{x_{vehicle}-x_{stopline}}{v_{vehicle}}$$

The vehicle may maintain its current speed rather than slow down should the current phase for the approach be red, and the vehicle is expected to arrive once the signal changes to green, as given by:

$$t_{red\;remaining}<\frac{x_{vehicle}-x_{stopline}}{v_{vehicle}}$$

### 3.4. Defining the Proposed System

**Figure 2:**
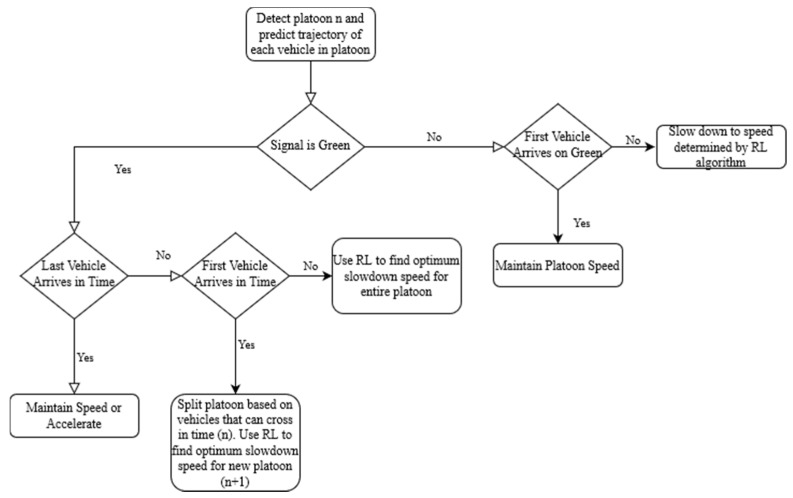
Flowchart depicting a simplified version of the algorithm.

Considering the idealized system, a mixed dynamic programming and reinforcement learning approach was selected. In order to facilitate the task and reduce the exploration space, a set of conditions were established in which the system would apply ideal speeds obtained using a reinforcement learning algorithm. A flowchart of the proposed system is shown in Figure 2.

Considering any given approach, the reinforcement learning algorithm would only activate if the system identified that a vehicle is unable to clear the intersection. Under these conditions, ideal slow-down speeds will be determined and applied to specific platoons in the approach. Simple solutions to the optimum behavior were identified, namely a situation where a vehicle may clear the intersection while traveling at maximum speed, and a situation where stopped vehicles prepare to clear the intersection as fast as possible. In the first situation, the simple solution would be not to slow down and to clear the intersection to allow for fewer vehicles to be stopped once the signal phase changes. Similarly, in a scenario where vehicles are not in motion or are moving at extremely low speeds (<5 km/h) at a red light, the ideal behavior would be to accelerate to the speed limit once the phase changes if it is safe to do so.

It should be noted that the algorithm deals with connected vehicles rather than autonomous vehicles. The speeds in question act as speed limits rather than actuated speeds. Consequentially, issuing a speed advisory of 50 km/h to a stopped vehicle informs a vehicle that it may travel up to 50 km/h rather than informing it to accelerate to that speed immediately and potentially hit a stopped vehicle in front of it. Thus, the working assumption is that within the speed distribution commonly observed when setting a speed limit, road users tend to accelerate to the maximum legal driving speed on average and have a propensity to prefer higher speeds.

### 3.5. Determining the Ideal Speed

The ideal speed of a platoon of vehicles at any given point in time, $v_{cr}$, is dependent on the current state. Depending on the situations outlined in previous sections, the vehicle may be asked to speed up or slow down. While it may be possible to develop a formula using optimization that would eventually integrate into an ATSC system, this would be too computationally intensive given the large number of computations performed for every vehicle in the system. Instead, a reinforcement learning approach was used, provided that the situation warrants a slowdown. Reinforcement learning (RL) approaches are functionally similar to linear regression models with regards to their relatively low computational intensity. RL approaches are thus superior to conventional approaches in that they can easily perform actions in real-time, and that they do not require site-specific formulation to function, instead opting for a largely data-driven approach to achieve great benefits to safety.

#### 3.5.1. Network Architecture

Given the constraints relating to real-time use, a reinforcement learning approach was considered to be the best way forward. Due to the continuous state and action space, a simple *Q*-learning or Deep-*Q*-Network approach was not feasible. As such, a model with a state space that may be discretized, and a continuous action space was selected. A DDPG architecture was thus used in this scenario and implemented using the MATLAB Reinforcement Learning Toolbox with three hidden layers of 100 neurons representing each of the actor and critic networks. A total of 46 input nodes and 8 output nodes, representing the input variables and the actions, respectively, were used.

DDPG agents use an actor and critic network in order to learn from estimated targets and upgrade the network to ensure stability. Overall, four neural networks were used including a *Q* network, a deterministic policy network, a target *Q* network, and a target policy network. Learning is characterized by maximizing the Bellman Equation, a formula that is used in reinforcement learning to describe the ideal action-state pair that optimizes for a given *Q* function. Since a DDPG approximates this *Q* function, it utilizes a mean-squared bellman error function that is to be minimized given by: [45,49]

$$L\left(\Phi ,D\right)=E_{\left(s,a,r,s^{\prime },d\right)\sim D}\;\left[{\left(Q_{\Phi }\left(s,a\right)-\left(r+\gamma \left(1-d\right)Q_{\Phi targ}\left(s^{\prime },{µ}_{\theta_{targ}}\left(s^{\prime }\right)\right)\right)\right)}^{2}\right]$$

A deterministic policy is selected that maximizes *Q*_*Φ* (s,a) via gradient ascent, as given by:

$$\underset{\theta }{\max}\underset{s\sim D}{E}Q_{\Phi }\left(s,{µ}_{\theta_{targ}}\left(s\right)\right)$$

Over time, the algorithm refines the *Q*-network by updating the various weights resulting in improved performance. While the model is deterministic in nature, it utilizes an Ornstein Uhlenbeck noise function to account for random exploration, while it exploits existing knowledge. The noise parameter used was assumed to be 10% of the total action space range with the predefined standard mean attraction rate of 0.15. Noise over time similarly decreases at a predefined rate provided that the Ornstein Uhlenbeck function is stable:

$$1\geq \left|1-{µ}_{attraction}*t_{sample}\right|$$

The reward function, arguably the most important part of a reinforcement learning algorithm, was selected to be the sum of shockwave areas, which is the main parameter used to approximate traffic conflicts. The shockwave area was used in lieu of the other formulas to utilize the platoon ratio. This was done since the safety performance functions obtained apply to entire cycles, and incremental changes in area cannot be represented as changes in conflicts given the significantly smaller timesteps. In theory, optimizing to reduce the shockwave area with several steps of look ahead would be similar to optimizing for the exponential function of shockwave area obtained from Essa et al. 2018 [3]. This assumption was subsequently verified by the performance metrics used in the evaluation.

$$E\left(Y\right)=-\overset{4}{\underset{App=1}{\sum }}\overset{Lane_{n}}{\underset{Lane=1}{\sum }}\;V^{0.706}exp\left(-1.797+0.501\;A\right)$$

#### 3.5.2. Defining the Action Space

The ideal speed, defined as the required speed to optimize the reward function in previous sections, is to be selected using a reinforcement learning DDPG approach. Every cycle, an unknown number of vehicles enter the intersection’s detection range with anywhere from approximately 1 to 300 vehicles being detected by the system at a time. Selecting an individualized speed for each vehicle, while more optimal, is extremely difficult computationally considering that each output value represents a discrete speed.

To ensure that this system is applicable to real-world conditions, a sensor range of 225 m from the stop line was assumed to be the maximum range at which vehicles may transmit and receive information. This figure was obtained from the average ranges of standard V2I DSRC systems, which vary from 150 m to 300 m [1]. As a result of this range, there would likely be at most three platoons in the intersection at any given point in time, with two platoons in motion and one stopped at the intersection. At all other points in time, a maximum of two moving platoons would likely be present given the spatial constraints.

Thus, for each approach, the leading vehicle speeds would be defined as $V_{platoon\;1}$ and $V_{platoon\;2}$, and would be applied as a desirable speed for all connected vehicles in the platoon. The range of allowable slowdown speeds that were selected were bounded between 30 and 50 km/h, further simplifying the action space. For an intersection with four approaches, this represents 8 possible actions that may be taken. Each discrete speed within this range was assigned a speed distribution with a similar variance to that of a typical speed limit, but with a shifted mean.

#### 3.5.3. Defining the Input Variables

The state of an intersection from cycle to cycle is continuous and a near infinite number of variables with countless combinations may be used. By examining the literature and observing key parameters, this continuous state space was discretized. Variables were obtained for each of the four approaches ensuring that said variables were readily available via V2V and V2I data. Thus, a set of 4 inputs was obtained for each variable, each representing the state of their respective approaches.

**Figure 3:**
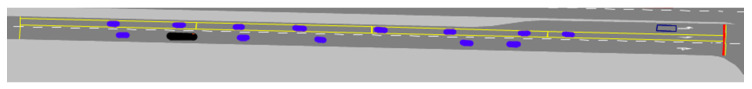
Graphical representation of the roadway in Vissim split into four parts on one lane (n1 = 1, n2 = 5, n3 = 4, n4 = 3).

The number of vehicles occupying a specific portion of the road was among the most important variables used. The number of vehicles would theoretically be key, as typically fewer vehicles on the road result in fewer traffic conflicts. However, with trajectory optimization, the distribution of vehicles would be equally as important. As such, the roadway may be split into four different segments, each with a certain number of vehicles, as shown in Figure 3. Thus, segments of 56.25 m were selected to represent occupancy and the spatial distribution of vehicles. While it would have been possible to further split the roadway into smaller segments, this would result in additional variables that may complicate the convergence process. In the example shown in Figure 3, the system would register one vehicle in Section 1, five in Section 2, four in Section 3, and three in Section 4, registering their respective speeds. By considering the spatial distribution of roadway occupancy, these variables help prevent the system from selecting actions that would adversely affect platoon length and, by extension, safety.

Average speed was also selected as a significant variable. While platoon speed could have been used, average speed allows the system to recognize where vehicles are slowing down and where they are accelerating. Average platoon speed would be restrictive with respect to allowing the system to combine or divide different platoons and would not be conductive towards identifying stop-and-go waves. A similar methodology was used to account for the spatial distribution of vehicle speeds. The same 4 roadway segments were utilized, and an average speed was obtained for each segment.

In order to compliment the spatial variables selected and to capitalize on the benefits of platooning, the gap between subsequent platoons was selected as an input variable. Given the sensor range of 225 m, there would typically be at most three platoons within range at any given point in time. For the algorithm, merging the platoons would require some degree of awareness about the distance between subsequent groups of vehicles. Thus, the gap between the first and the second platoon of vehicles was selected as a key variable. If a second or third platoon does not exist, the distance between the last detected platoon and the sensor range is assumed to be the platoon gap to overcome the limitations of the sensor range. The system assumes that if there is no detected platoon in range, a platoon will arrive shortly thereafter.

Temporal variables are equally important in trajectory optimization. Neural networks typically do not work well with binary variables as during the backpropagation process, and in the computation of errors, fractions or strange weights may arise. To overcome this limitation, rather than represent the signal states as a binary value, the current time elapsed since a given phase is a superior measure to inform the system about how much time is left in the current signal phase on a given approach. Based on this information, higher or lower speeds may be selected depending on whether there will soon be a phase change. Consequentially, the time since the last red and time since the last green were used as temporal variables.

It should be noted that the system optimizes all four approaches simultaneously. As such, it may be difficult for the system to identify which variable corresponds to which approach resulting in some non-existent correlations being erroneously detected. A collaborative multi-agent approach may be used to allow for four DDPG agents to simultaneously optimize each approach; however, this may complicate matters when adding additional dimensions to the problem, such as signal optimization. A simpler solution is to allow the output of the precedent action to be represented as an input to the following computation. This allows for a single agent system to be able to better identify the rewards resulting from a given action on a given approach. The explanatory variables selected as inputs to the model are detailed in Table 1.

## 4. Validation and Testing

### 4.1. Modeling the Environment

The proposed model was applied to a simulation to determine the potential benefits of the system. Vissim simulation software was used to model the effects of this framework on a real intersection. Data from two intersections, 128th Street and 72nd Avenue and 132nd Street and 72nd Avenue in Surrey, BC, were utilized to create and calibrate a Vissim model and ensure that it is an accurate representation of reality [3].

The modeling environment, PTV Vissim, utilizes a Wiederman 99 car-following model. The model, which accurately represents car following behavior, allows for the creation of several different vehicle classes. A custom class relating to connected vehicles was created that allows for all V2I and I2V communications to be limited to vehicles of the CV class.

PTV Vissim’s COM integration allows for the extraction of real-time traffic data from the simulation, including position, speed, and other relevant parameters and dynamic assignment of desired speed distributions. Different parameters may also be changed during a simulation, including signal states as well as individual kinematic variables for specific vehicles. Of note is the desired speed of a vehicle, which may be modified during the simulation. The desired speed represents a predefined speed distribution that defines a vehicle’s acceleration and deceleration behavior. This may be used to emulate speed advisories given that the behavior is similar to driver reaction to speed limits, and slight variations in speeding/slowing down behavior are likely to be observed in practice. Speed distributions were created in PTV Vissim emulating the typical distribution of vehicle speeds at the speed limits of 30, 40, and 50 km/h with a shifted mean to represent desired speed distributions for discrete speed limits within this range (e.g., centered at 32, 36, 42, 49 km/h).

### 4.2. Defining the Test Sites

A total of two intersections were modeled with varying scenarios created to represent different arrival patterns and traffic volumes throughout the day. The first intersection on 128th Street and 72nd Avenue was used to train the reinforcement learning model, while the second intersection was set aside to be used to validate the results.

The first intersection, located on 128th Street and 72nd Avenue in Surrey, BC, is comprised of four approaches, each with two through lanes and a left turn lane at the intersection. A bus route is present on 72nd Avenue. A loop detector system, which allows for adaptive traffic signal control using left turn vehicle occupancy, was also present on all four approaches. This was considered as the main test site, representing a typical intersection. The algorithm’s performance on this intersection was considered to be representative of standard conditions for two-lane roads.

**Figure 4:**
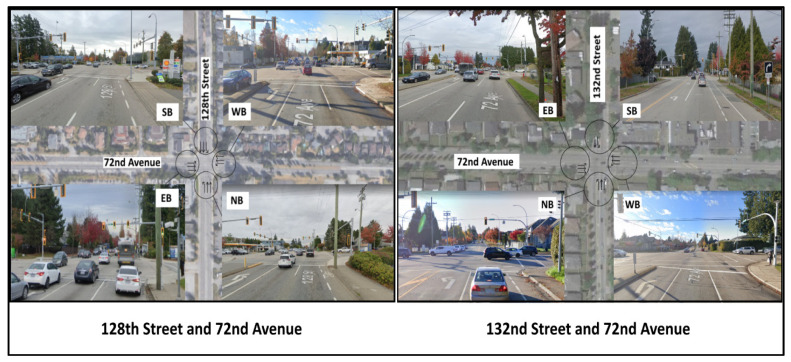
The two intersections: 128th Street and 72nd Avenue, and 132nd Street and 72nd Avenue.

A second intersection located on 132nd Street and 72nd Avenue in Surrey, BC (Figure 4) was selected to validate the algorithm due to its distinct characteristics. Unlike the first intersection, this intersection features a southbound approach with one lane and a -left-turning bay, as well as a northbound approach with a right-turning lane, a through lane, and a -left-turning bay. The eastbound and westbound approaches are similar to that of the first intersection, with two through lanes and a left turn lane on each approach. Detector loops and bus routes were present on all four approaches of this intersection. These differing characteristics allow for the algorithm to be tested on various different geometric and temporal conditions that will provide insight into the effectiveness of the algorithm on different types of roadways. An additional benefit of using this as validation would be to observe the effects of transferring a trained model onto a new intersection and observing the effect of training.

**Figure 5:**
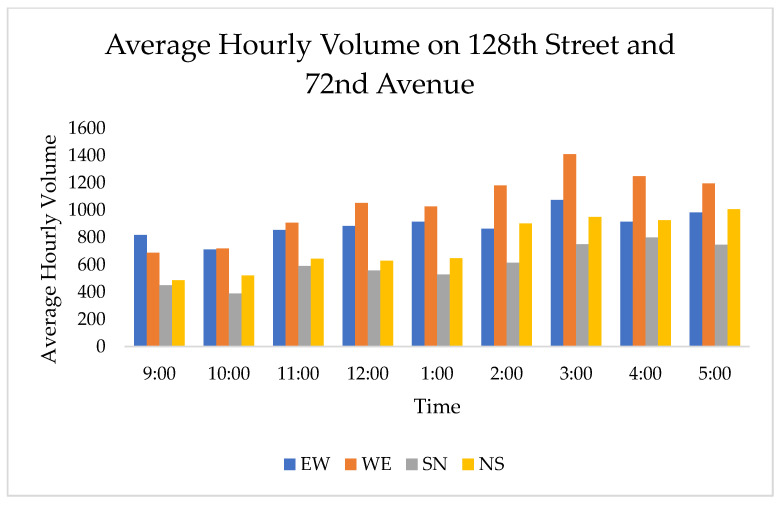
Hourly average traffic volumes on 128th Street and 72nd Avenue.

**Figure 6:**
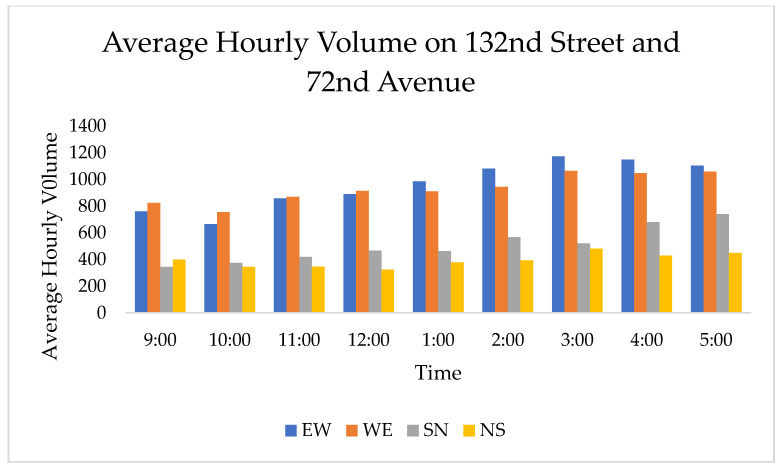
Hourly average traffic volumes on 132nd Street and 72nd Avenue.

The hourly traffic volumes observed on 128th Street and 72nd Avenue ranged from 2343 vehicles at 10:00–11:00 a.m. to 4186 vehicles at 3:00–4:00 p.m., and the hourly traffic volume observed on 132nd Street and 72nd Avenue ranged from 2140 vehicles at 10:00–11:00 a.m. to 3354 vehicles at 5:00–6:00 p.m. It should be noted that the southbound and northbound traffic volumes are for a single lane of traffic, which differs from the two-lane northbound and southbound approaches on 128th Street. The hourly traffic volume data are shown in Figure 5 and Figure 6.

### 4.3. Training Process

The first intersection at 128th Street and 72nd Avenue in Surrey, BC, was used to train the model. Episodes, which consisted of random 20-min samples from simulation hours, were used in the training process during which the model’s performance was expected to improve over time. The model was initially trained based on traffic conditions that represent the scenario between 12:00 p.m. and 1:00 p.m., before being applied to random hours between 9:00 a.m. and 5:00 p.m. This was done to generalize the training dataset as to not overfit to a specific traffic volume. Over 450 episodes were run, with a minimum of 50 episodes per simulation hour being performed for each scenario. While simulation time may vary based on the device used, each episode took approximately an average of 8 min to complete, for a total approximate training time of 60 h.

Each simulation episode consisted of several timesteps with a defined Δt of 5 s. The trajectories of vehicles were obtained every second in real-time. These trajectories were used to update a trajectory matrix every Δt, which was used to obtain both the input parameters to the RL model (occupancy, speed, platoon gap, etc.) as well as predict future trajectories. The system thus considers these future trajectories when updating the platoon speed and relays speed advisories to the vehicles in question through the use of desired speed distributions.

Vissim random number generator (RNG) seeds were used to account for the inherent randomness in the system to overcome limitations within the model and avoid overfitting using the DDPG RL algorithm. The Ornstein Uhlenbeck noise parameters were similarly selected as to avoid repeatedly applying the same “optimum policy” to the same situation without exploring potentially better solutions and updating the policy based on new states observed.

Once the system was trained on the intersection at 128th Street and 72nd Avenue for 450 episodes, baseline safety performance was established based on fixed seeds throughout the day. This established the basis for which model performance could be compared. The process was repeated for the second intersection resulting in the creation of a “do-nothing option” scenario where baseline safety was quantified.

### 4.4. Testing Procedure

After 450 episodes of training, the model was run for an additional 100 episodes to obtain an average number of conflicts per approach. These results were then compared to the baseline simulations, where no actions were being taken.

To ensure transferability, a secondary intersection at 132nd Street and 72nd Avenue was used to validate the model and ensure that the benefits are indeed transferable without additional training and applicable to approaches with different characteristics.

Initially, the model was run assuming that all vehicles on the road are CVs that comply with speed advisories. It is not practical to assume 100% MPR in the near future, and hypothetically, even if it is achieved, compliance remains an issue. However, this scenario represents the maximum possible improvement as a result of using this system. Alternate scenarios relating to a mixed environment were created and tested. Scenarios with 100%, 75%, 50%, 25%, 15%, and 10% market penetration ratios were tested to examine the effects of MPR and, by extension, low compliance. Conventional vehicles are assumed to be invisible for the purpose of the RL algorithm inputs and do not receive speed advisories. They are nonetheless considered in the final computation of traffic conflicts. MPRs under 10% were not tested due to a high expected variance, which would be more reflective of the simulation environment than real-world conditions. Thus, 10% MPR was considered to be reliable enough to be used to estimate system performance at lower levels of MPR.

The performance over 100 trial runs was observed for each scenario, and the distribution of results were compared based on conflict reduction to evaluate performance. Given that each intersection consists of four different approaches and the system was tested on two intersections, the system was tested on a total of eight different independent approaches, each with six different fleet compositions. Since the system tests an individual controller for all four approaches of an intersection, the overall net safety benefit was considered in the computation of the results rather than individual benefits to each approach. Randomness during the training process was accounted for through the use of the initial RNG seed built into the Vissim simulation as well as a series of dummy intersections simulating an arrival pattern representative of the study intersections.

The training process was created based on expected real-world behavior. Given that the system utilizes a DDPG algorithm to train the model, there is a large amount of inherent randomness in the first few sets of exploratory actions. As such, a completely untrained model may perform poorly in practice and result in additional traffic conflicts that could have been prevented. To remedy this, the intent of this training process was to create a model that may be trained on a real or simulated intersection and could then be transferred onto another similar intersection to achieve some safety benefits at the new intersection without additional training. Additional training would then further improve safety at that intersection over time as the system learns more intersection-specific patterns and characteristics as they relate to safety.

## 5. Results

### 5.1. Quantifying Safety at Full Implementation

After running hundreds of simulations, the algorithm managed to yield an average conflict reduction of 23% when applied to 128th Street and 72nd Avenue. The overall results were promising, with most trials seeing a statistically significant improvement to safety ranging from a minimum daily conflict reduction of −2% to a maximum of 38% when considering the sum of best and worst-case scenarios for every hour. The statistical parameters are shown in Table 2 and represent the total sum of hourly conflicts compared to the total sum of hourly baseline conflicts between 9:00 a.m. and 5:00 p.m.

Judging from these results, the implementation of this algorithm at full market penetration without considering additional benefits as a result of other technologies available with CVs and CAVs yields considerable results. On an hourly basis, an improvement to safety in any given hour may be expected 88% of the time. When considering the sum of worst-case scenarios for every hour, a daily increase in traffic conflicts of 2% may be expected, indicating that any negative effects observed in one given hour will likely be offset by benefits in another.

**Figure 7:**
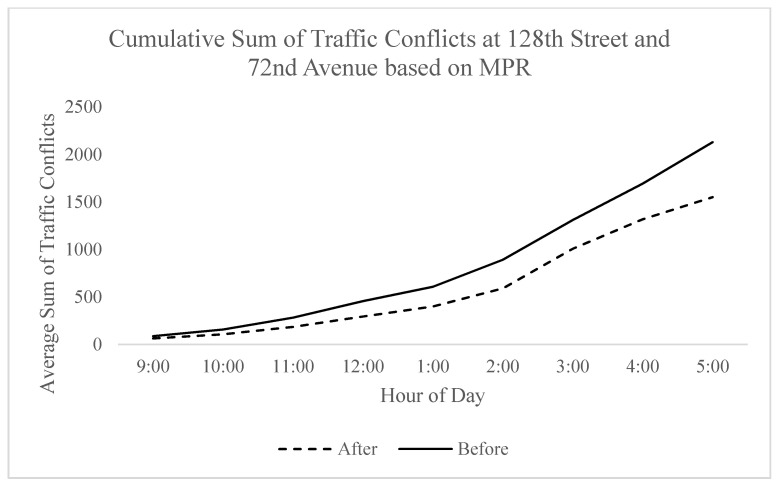
Variation of conflict rate per hour on 128th Street and 72nd Avenue.

The algorithm was tested on the test intersection over the course of a day with traffic conditions between 9:00 a.m. and 5:00 p.m. being modeled in Vissim. The results ranged from a significant reduction in hourly traffic conflicts to negligible reductions based on the time of day. Initially, a compensation effect was observed where approaches with less exposure would achieve comparably less absolute improvement due to there being fewer conflicts to prevent. However, as the traffic volume increased, greater improvements were observed. This was highly dependent on the arrival patterns in which certain more favorable patterns resulted in greater conflict reduction, as seen in Figure 7. Traffic volume did not seem to have a considerable effect on percent conflict reduction.

### 5.2. Impact of MPR on Safety

**Figure 8:**
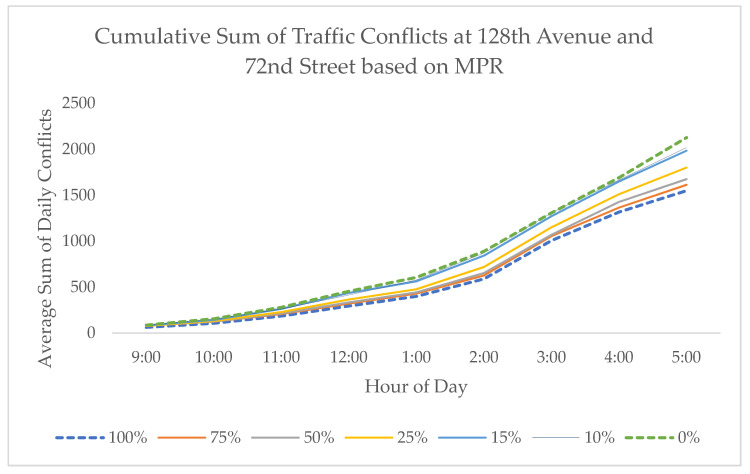
The effect of market penetration rate on conflict rate.

Test scenarios for the 100%, 75%, 50%, 25%, 15%, 10%, and 0% MPR scenarios were tested on 128th Street and 72nd Avenue and yielded positive results as shown in Figure 8. Similar to previous studies, the bulk of the safety benefits begin to manifest themselves when approximately a quarter of vehicles on the road are CVs, with the largest jump in conflict reduction observed between 15% and 25% MPR where 74% of the total benefits are attained. At 10% MPR, a 6% total reduction in conflicts can be observed, indicating that the system can improve safety early in the CV implementation process.

This indicates that this system is functional in the transitional state when not all vehicles on the road are connected and thus is applicable in the near future. However, certain outliers were observed, as seen in Figure 8, which resulted in a higher MPR corresponding to increased conflicts at lower levels during certain times of the day. This indicates that the system may work in most scenarios but underperforms under certain conditions. However, a compensating effect is observed when considering the entire dataset, which offsets the negative effects.

### 5.3. Validation and Transferability

To ensure that the system is transferable, the same algorithm was applied to 132nd Street and 72nd Avenue without additional training. The roadway characteristics of 132nd Street and 72nd Avenue are different, including two one-lane through approaches as opposed to two-lane approaches and a far larger left turn bay and left-turn volume.

Without training and including all approaches, the total daily improvement to safety was 9% and varied greatly throughout the day. The system reduced an average of 61 conflicts per hour, with a peak conflict reduction of 412 conflicts at 3:00 p.m. and mixed performance throughout the day. When comparing the intersections and controlling for volume, the peak conflict reduction at 132nd Street and 72nd Avenue represented a 28% reduction at 3:00 p.m. when compared to a peak reduction of 42% at 5:00 p.m. on 128th Street and 72nd Avenue. The average daily reduction in traffic conflicts on 128th Street and 72nd Avenue and 132nd Street and 72nd Avenue were 23% and 9%, respectively. This indicates that the algorithm yielded positive values on the validation intersection but not to the same degree as the benefits seen on the test intersection.

**Figure 9:**
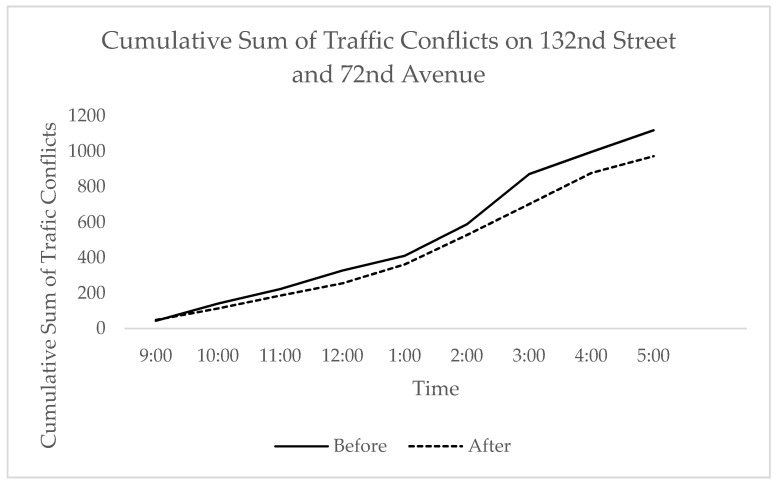
The effect of market penetration rate on conflict rate.

The disparity in the benefit may have been attributed to the difference in geometric characteristics of the various approaches. In order to ensure that an appropriate comparison was made, the results were filtered to only account for similar approaches (those on 72nd Avenue). As a result, a total average conflict reduction of 15% was observed, as shown in Figure 9 and Table 3.

Despite the different geometric and temporal characteristics when considering 132nd Street, it can be said that the proposed system would result in a net improvement in road safety without training. With additional training, the benefit would be significantly greater, and similar effects may be observed. However, this serves as an important measure to visualize what would happen if the trained dataset was not fully representative of the spatial conditions at another intersection. This is not to say that this algorithm is ineffective in other intersections but highlights the importance of site-specific algorithm training and conflict reduction measures.

## 6. Discussion

### 6.1. Observed Vehicle Behavior

**Figure 10:**
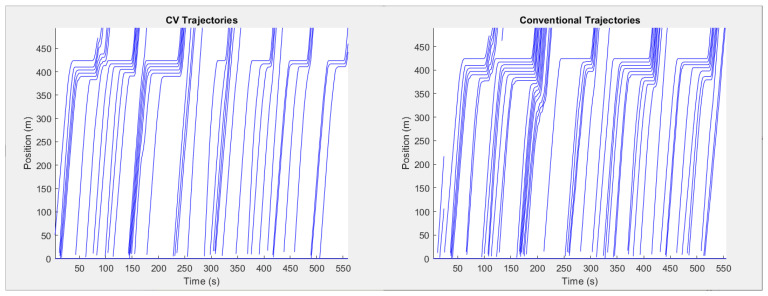
Side-by-side comparison of space-time diagrams for the Westbound Approach on 72nd Avenue (132nd Street and 72nd Avenue) (stop line at x = 425 m).

The proposed algorithm demonstrated behavior that was similar to the ideal behavior previously discussed, with vehicles slowing down such that they may arrive at the intersection in time for the next green while avoiding the creation of stop-and-go waves or unnecessary delays. The space-time diagram in Figure 10 demonstrates this relationship where a smaller shockwave area was observed over eight cycles due to the algorithm’s ability to dissipate shockwaves by controlling individual vehicle speeds. It should be noted that this represents the trajectory observed on a single lane, and that lane changing behavior may influence the visualization creating the discontinuities observed in Figure 10.

This behavior also applies to scenarios where the market penetration level was lower than 100%, in which case the effectiveness of the slowdown commands was dictated primarily by the probability that the leading vehicle of a platoon of vehicles is a connected vehicle. In situations where the leading vehicle or subsequent vehicles were not connected, the benefit was not seen, and the shockwave was not as effectively dissipated. This indicates that while the full implementation condition results in consistent safety benefits, the intermediate state would see significantly greater fluctuations in conflict reduction. At 100% MPR, the system appears to control the queue length without the need to set static upper bounds. In most cases, this prevents the formation of additional shockwaves upstream.

It is worth further analyzing the performance of 132nd Street, which did not perform as well as 128th Street. This is largely attributed to the differences based on the different types of approaches. On the eastbound and westbound approaches of 132nd Street and 72nd Avenue, the algorithm performed with comparable performance to 128th Street and 72nd Avenue due to the similar characteristics. A similar variance was also observed.

Further analyses were thus performed on the northbound and southbound approaches, which had different constraints. Upon further observation, the bulk of the increase in traffic conflicts resulting from the implementation of the algorithm stems from the southbound approach. Since there is only one lane, right-turning vehicles, which may turn right on red, may reduce traffic flow if they reduce their speeds at a red light. Given the shorter signal time, these vehicles and slower moving traffic, in general, cause an accumulation of vehicles. Fewer vehicles are able to clear the intersection per cycle, resulting in increased conflicts due to longer queues. This offsets benefits gained on other approaches. Similarly, left-turning vehicles seeking to change lanes into the left turning bay may slow down these right-turning vehicles, a phenomenon not observed on the other approaches due to the ability of vehicles to change lanes and the significantly smaller right-turning volume from the minor road.

In contrast, this result was not seen on the northbound approach as right-turning vehicles merged into the right-turning lane and thus did not unnecessarily slow down following vehicles. The individual impact of this approach was estimated to have resulted in a 22% reduction in conflicts relative to the baseline when compared to the southbound approach, which resulted in a 34% increase in conflicts, offsetting the greater gains to safety elsewhere. These effects were further affected by the difference in traffic volumes on each approach.

The implementation of this system is possible and recommended if prior studies are performed using data specific to the intersection in question. The algorithm functions well in both high volume and low volume conditions provided that there are multiple lanes available and the right-turning traffic is either exclusive, protected, or small relative to the through volume. Blind or inappropriate implementation of this algorithm, as with any other safety-related intervention, may worsen the situation if implemented poorly. Nonetheless, the results show benefits in multilane intersections with lesser benefits on single-lane roads. Thus, as with any intervention, the system should be implemented based on a site-by-site analysis with simulations and studies testing the efficacy of the algorithm prior to implementation, with potential for large reductions in rear-end traffic conflicts. The reinforcement learning aspect of the algorithm must be trained on the intersection in question as is evident in the recorded data. Should additional training be performed, the results are expected to drastically improve, and as such, different parameters related to roadway geometry, arrival patterns, vehicle compositions, and volume that are site-specific should be considered in the training model.

### 6.2. Market Penetration Rate and Implications for Policy

The proposed algorithm provides a low-cost approach that is not computationally intensive and works towards optimizing for safety by reducing rear-end traffic conflicts. The conflict reduction increases with increased MPR with diminishing returns. A sum of 74% of the total benefit can be obtained when one in four vehicles has I2V and V2I capabilities, not including further benefits resulting from the proliferation of connected vehicles.

While an average conflict reduction of 15–23% may be expected as a result of trajectory optimization alone, further improvements to safety may result from other factors such as the use of CVs in adaptive traffic signal control systems, as well as other systems which utilize CV data and CAV technologies to improve safety. Such systems at low levels of automation that would proliferate alongside CVs may feature assisted braking and lane assist systems, which would further improve safety. The most significant intervention that may help further improve safety is ATSC systems. ATSC systems may be used to dynamically optimize traffic signals to help dissipate shockwaves that form due to slowed or stopped traffic. When combined with trajectory optimization, significant safety benefits are likely to be observed.

## 7. Conclusions

This paper presented a real-time trajectory optimization algorithm that utilized real-time data obtained from V2I sensors located on connected vehicles. The data were analyzed using a hybrid dynamic programming algorithm, which uses a DDPG reinforcement learning agent to minimize the shockwave area and thus reduce rear-end conflicts.

In particular, the DDPG reinforcement learning algorithm utilized shockwave area as a surrogate for conflict rate and then computed the conflict rate to verify this assumption. The continuous state space was discretized and split into key variables relating to occupancy of certain sections of the road, speed of vehicles occupying a certain roadway section, the gap between platoons, and signal timing parameters. Subsequently, these discrete inputs were considered by the algorithm, which provided vehicles with a real-time speed advisory and returned a near-instantaneous real-time reward based on the shockwave area.

The algorithm was trained using PTV Vissim simulation software, making use of its COM feature to send commands to modify each individual vehicle’s desired speed during the simulation at time steps of Δt = 5 s. A total of eight different speeds were decided upon during each timestep once the simple solutions, maintaining the same speed or accelerating to the speed limit, were eliminated from the action space.

The algorithm was evaluated with respect to conflict reduction and yielded a notable daily conflict reduction of 23% on average and ranged from 2% to 37% on the first study intersection. On the secondary intersection with different characteristics, a conflict reduction of 9% was observed with adverse effects on safety being observed on the southbound single-lane approach, which did not have an exclusive right turn lane. The data were then filtered to ignore approaches with significantly different characteristics, and a conflict reduction of 15% was observed. A further study into the effect of market penetration rate demonstrated that the majority of the benefits obtained as a result of this system can be obtained at relatively low MPRs such as at 25%. This has policy implications with respect to the promotion of CVs and planning new intelligent transportation system infrastructure.

Additionally, further improvements to the system and the potential for synchronization with ATSC systems were discussed. All in all, this paper presented a simple V2I/I2V based framework using reinforcement learning to improve safety in real-time. The benefits of this algorithm, should it be implemented, are set to increase with time as both computing power and market penetration rate improve. This would consequentially allow for more complex algorithms to be devised and further reduce conflicts, perhaps considering other conflict types, such as side swipes and crossing conflicts.

Despite the algorithm’s effectiveness, several limitations were identified. Firstly, as noted by Matowiki et al. 2016 [31] in the trial of variable speed limits, it is entirely possible that human drivers would opt to ignore the speed advisories in favor of a perceived personal benefit rather than a system optimum solution, potentially endangering themselves or other drivers. Additionally, the system assumes that certain “pacer vehicles” that are connected may work to slow down and regulate the speed of conventional vehicles behind them. It is possible that these vehicles would face social pressure to speed up despite the warnings provided to them that they will not arrive at the intersection in time. As a result, dangerous lane-changing behavior to pass the connected vehicles may be observed. Thus, the implementation of the said system would likely require some degree of education to the public to understand that slowing down and obeying the recommendations will result in shorter wait times and a safer drive.

Another limitation to the study was that this model did not consider sensor malfunctions or response time and assumed that all parts of the process including detection are instantaneous. A simple solution exists to address this concern. If the reaction time and sensor time are defined as Δt, it would be possible to estimate a vehicle’s position at time t using basic kinematic equations and thus “offset” the model by a few seconds and extend the refresh rate. This would slightly alter the final results, and system resilience should be re-examined in future studies. According to the literature, delays in AV and CAV environments may result in significant traffic instability [50]. However, given that this paper evaluates a CV environment that does not have actuated control by the controller, this effect is unlikely to be seen. In the event that a V2I sensor malfunctions and transmits corrupt or faulty data, the data-driven approach used by the controller may provide vehicles with a suboptimal solution as a result. A built-in safeguard for this scenario is the other variables considered, which may, nonetheless, prove to be appropriate predictors for the model. For example, if the number of vehicles in one quadrant is erroneously underreported, the speed of vehicles or the gap between platoons may overcome the inaccuracies and result in a simply sub-optimal decision rather than a poor decision. Despite the benefits that are likely to be observed in implementing this algorithm, one should recognize that all systems have flaws and additional work should be performed in creating redundancies, especially when dealing with traffic safety and human drivers.

Lastly, the devised algorithm worked to reduce rear-end collisions and did not consider other conflict types such as side-swipe conflicts or crossing conflicts. These conflicts were assumed to be constant and unaffected by the algorithm. In future studies, this assumption should be verified, and a more sophisticated algorithm should be devised that would consider alternative conflict types.

## Figures and Tables

**Table 1:** Relevant input variables considered by the reinforcement learning algorithm.

Variable Name	Definition	Variable Name	Definition
n11	Number of Vehicles on Approach 1 Occupying Region 1	v32	Average Speed of Vehicles on Approach 3 Occupying Region 2
n12	Number of Vehicles on Approach 1 Occupying Region 2	v33	Average Speed of Vehicles on Approach 3 Occupying Region 3
n13	Number of Vehicles on Approach 1 Occupying Region 3	v34	Average Speed of Vehicles on Approach 3 Occupying Region 4
n14	Number of Vehicles on Approach 1 Occupying Region 4	PGap3	Gap between first and second platoon on Approach 3
v11	Average Speed of Vehicles on Approach 1 Occupying Region 1	n41	Number of Vehicles on Approach 4 Occupying Region 1
v12	Average Speed of Vehicles on Approach 1 Occupying Region 2	n42	Number of Vehicles on Approach 4 Occupying Region 2
v13	Average Speed of Vehicles on Approach 1 Occupying Region 3	n43	Number of Vehicles on Approach 4 Occupying Region 3
v14	Average Speed of Vehicles on Approach 1 Occupying Region 4	n44	Number of Vehicles on Approach 4 Occupying Region 4
PGap1	Gap between first and second platoon on Approach 1	v41	Average Speed of Vehicles on Approach 4 Occupying Region 1
n21	Number of Vehicles on Approach 2 Occupying Region 1	v42	Average Speed of Vehicles on Approach 4 Occupying Region 2
n22	Number of Vehicles on Approach 2 Occupying Region 2	v43	Average Speed of Vehicles on Approach 4 Occupying Region 3
n23	Number of Vehicles on Approach 2 Occupying Region 3	v44	Average Speed of Vehicles on Approach 4 Occupying Region 4
n24	Number of Vehicles on Approach 2 Occupying Region 4	PGap4	Gap between first and second platoon on Approach 4
v21	Average Speed of Vehicles on Approach 2 Occupying Region 1	TSG	Elapsed Time Since Green Phase on Approach 1
v22	Average Speed of Vehicles on Approach 2 Occupying Region 2	TSR	Elapsed Time Since Red Phase on Approach 1
v23	Average Speed of Vehicles on Approach 2 Occupying Region 3	A11	Previous Action representing the input speed for Platoon 1 on Approach 1
v24	Average Speed of Vehicles on Approach 2 Occupying Region 4	A12	Previous Action representing the input speed for Platoon 2 on Approach 2
PGap2	Gap between first and second platoon on Approach 3	A21	Previous Action representing the input speed for Platoon 1 on Approach 2
n31	Number of Vehicles on Approach 3 Occupying Region 1	A22	Previous Action representing the input speed for Platoon 2 on Approach 2
n32	Number of Vehicles on Approach 3 Occupying Region 2	A31	Previous Action representing the input speed for Platoon 1 on Approach 3
n33	Number of Vehicles on Approach 3 Occupying Region 3	A32	Previous Action representing the input speed for Platoon 2 on Approach 3
n34	Number of Vehicles on Approach 3 Occupying Region 4	A41	Previous Action representing the input speed for Platoon 1 on Approach 4
v31	Average Speed of Vehicles on Approach 3 Occupying Region 1	A42	Previous Action representing the input speed for Platoon 2 on Approach 4

**Table 2:** Projected safety benefits on 128th Street and 72nd Avenue.

Parameter	Value
Average Hourly Conflict Reduction	23%
Median Hourly Conflict Reduction	20%
Sum of Maximum Hourly Conflict Reduction (Best Case Scenario)	38%
Standard Deviation	10%

**Table 3:** Variation of conflict rate per hour on 72nd Avenue (EB and WB approaches).

	Entire Intersection	EB and WB Approaches
Average Hourly Conflict Reduction	9%	15%
Standard Deviation	9%	9%

## Data Availability

Not applicable.
